# Carbonic Anhydrase Sensitivity to Pesticides: Perspectives for Biomarker Development

**DOI:** 10.3390/ijms21103562

**Published:** 2020-05-18

**Authors:** Maria Giulia Lionetto, Roberto Caricato, Maria Elena Giordano

**Affiliations:** Dept. of Biological and Environmental Sciences and Technologies (DiSTeBA), University of Salento, Via prov.le Lecce-Monteroni, 73100 Lecce, Italy; roberto.caricato@unisalento.it (R.C.); elena.giordano@unisalento.it (M.E.G.)

**Keywords:** carbonic anhydrase, biomarker, pesticides, herbicides, fungicides, enzymatic inhibition, protein adducts

## Abstract

Carbonic anhydrase (CA) is a widespread metalloenzyme playing a pivotal role in several physiological processes. Many studies have demonstrated the in vitro and in vivo sensitivity of CA to the exposure to several classes of pesticides in both humans and wildlife. This review aims to analyze and to discuss the literature available in this field, providing a comprehensive view useful to foresee perspectives for the development of novel CA-based pesticide biomarkers. The analysis of the available data highlighted the ability of several pesticide molecules to interact directly with the enzyme in humans and wildlife and to inhibit CA activity in vitro and in vivo, with possible alterations of key physiological functions. The analysis disclosed key areas of further research and, at the same time, identified some perspectives for the development of novel CA-based sensitive biomarkers to pesticide exposure, suitable to be used in several fields from human biomonitoring in occupational and environmental medicine to environmental monitoring on non-target species.

## 1. Introduction

Carbonic anhydrase, catalyzing the reversible hydration of CO_2_ to HCO_3_^-^ and H^+^, is a widespread metalloenzyme with its eight genetically distinct families: α-CA expressed in animals and algae, β-CA in plants and prokaryotes, γ-CA in archaea, δ-CA, ζ-CA and θ-CA expressed in marine diatoms, η-CA in protozoa, ι-CA in diatoms and prokaryotes [1,2,3].

Due to the central role of bicarbonate, protons, and CO_2_ in several physiological processes, CA is of pivotal importance in a number of functions. In animals, it is crucial in respiratory gas exchange, acid-base regulation, fluid secretion, metabolism, calcification, bone resorption, signal transduction, and cellular defenses against oxidative stress [4,5]. 

The animal αCAs show an active site, conically shaped, with a zinc atom at the base coordinated by a water/hydroxide ion and three histidines (His94, His96, His119). During catalysis, first, the Zn^2+^ bound hydroxide exerts a nucleophilic attack on CO_2_ producing zinc-bound bicarbonate that, in turn, is displaced by a water molecule [6]. Then, the Zn^2+^ bound hydroxide is regenerated by the proton transfer from the zinc-bound water molecule to the bulk solvent facilitated by the His64 residue acting as a proton shuttle. 

This catalytic process is sensitive to inhibition by several agents. In the last decades, the research on CA inhibition has experienced a great impulse resulting in the discovery and synthesis of a number of compounds useful for therapeutic purposes [7,8].

On the other hand, several chemicals relevant to environmental pollution have proven to inhibit the catalytic activity of carbonic anhydrase. Among these, a number of works have demonstrated the sensitivity of CA to pesticides both in humans and wildlife. However, a comprehensive view of this topic is lacking in the literature. 

Pesticides are widely used in agriculture, public health control, domestic environment for the control of a large variety of pests, but at the same time, their broad use raises concern about the risks for human health and the environment. 

Humans are exposed to pesticides through occupational or environmental exposure. Workers in the agricultural sectors or in pesticide production are the groups mainly exposed to these compounds. The general population is exposed to pesticides and their degradation products indirectly through water, air, food, and dust, generally resulting in a low-level and long term exposure [9]. Moreover, pesticide run-off from agricultural lands and the subsequent release into water bodies further increases the dispersion of pesticides in the environment and, in turn, increases the probability of exposure of nontarget organisms in wildlife [10].

Over the last years, a great number of epidemiological studies have found significant relationships between the exposure to pesticides (via inhalation, ingestion, dermal contact, or across the placenta) with cancer, neurodevelopmental alteration in children, allergies, decreased fertility, and birth defects [11] in humans. In parallel, a number of ecotoxicological studies have demonstrated a wide array of negative effects on nontarget organisms in wildlife [12].

Pesticides can produce adverse effects, with a variety of alterations at the molecular, cellular, or tissue level, that can be used as biomarkers of exposure/effects in occupational and environmental medicine as well as in environmental toxicology studies [13]. 

Pesticide biomarkers are defined as molecular and cellular alterations in the human body or in a nontarget organism in response to pesticide exposure and can be useful for monitoring the presence of a chemical in the body, for detecting biological responses or assessing adverse health effects following exposure. Biomarkers of exposure detect the exposure of an organism to a chemical or mixture of chemicals. They can provide evidence of the route, pathway, and even the source of exposure; moreover, they can be useful for assessing the extent of exposure, its variations over time and among different populations. They can be represented by the direct measurement of the chemical of interest or its metabolites in the body fluids or can consist in an endogenous response reflecting the interaction of the compound with a subcellular target, such as the genesis of DNA or protein adducts detectable in the blood [13,14,15,16,17]. On the other hand, biomarkers of effect provide an assessment of toxicological effects in the organism, such as measurable biochemical, physiological, or behavioral alterations that can be directly related to the risk of adverse health effects. Biomarkers of susceptibility are represented by intrinsic characteristics of an organism that confers greater susceptibility to the adverse effects of exposure to a specific chemical. Clear examples are represented by polymorphisms of relevant xenobiotic-metabolizing enzymes [13].

The risk assessment and prevention of pesticide exposure are complex processes in relation to several factors such as, for example, the variations in the time and concentration of exposure, differences in the chemical structure and toxicity of the different classes of pesticides, mixtures of chemicals used, climate variations in the areas where the chemicals are used [18]. Therefore, the development of novel pesticide biomarkers is a growing need for improving the risk assessment process. 

This review aims to analyze and discuss the literature produced in recent years on the sensitivity of CA to a wide range of pesticides in order to foresee perspectives for the development of novel pesticide biomarkers suitable for human and environmental biomonitoring.

## 2. In Vitro Sensitivity of CA Activity to Pesticides in Animals 

Most of the studies available on the sensitivity of CA to pesticides in animal species come from in vitro experiments. Several classes of pesticides have been investigated for their inhibitory potential on CA activity in different species, most vertebrates, mainly fish and mammals. In particular, fish are often non-target organisms for the toxic action of pesticides, which represent one of the major pollutants for the aquatic environments due to the run-off from agricultural lands. The in vitro sensitivity of CA in fish and mammals (including humans) has been detected mainly in erythrocytes, liver, and gills. Erythrocytes are provided with a high CA activity involved in blood CO_2_ transport and excretion, the liver is equipped with different CA isoforms involved in metabolic processes and antioxidant defence [19,20], and gills possess abundant CA activity involved in the hydration of CO_2_ to produce H^+^ and HCO_3_^-^ needed for the branchial ion transport processes that sustain systemic ionic and acid-base regulation [21].

### 2.1. In Vitro Effect of Different Classes of Pesticides on CA Activity

**Figure 1:**
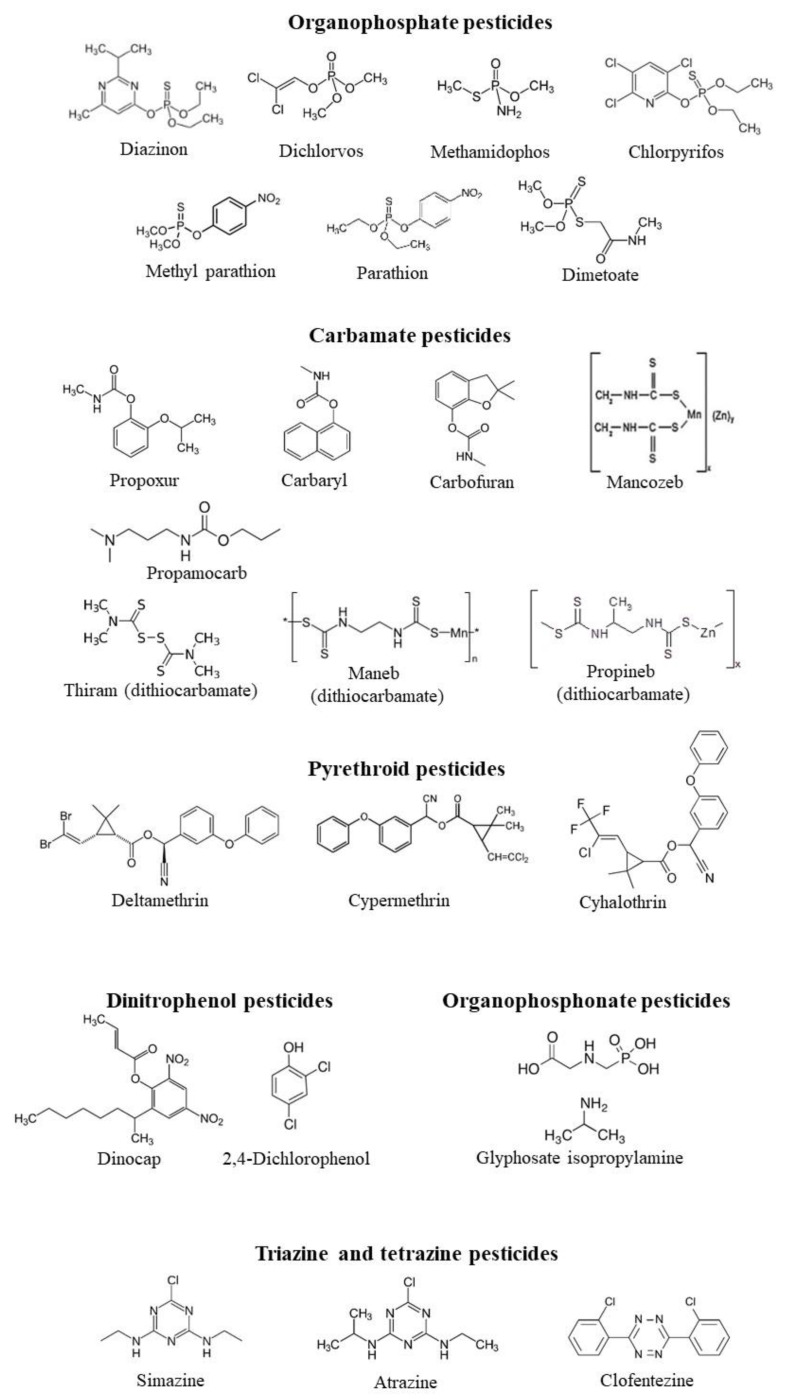
The chemical structure of the pesticides analyzed in Table 1 belonging to organophoshates, carbamates, pyrethroids, organophosphonates, dinitrophenol pesticides.

**Figure 2:**
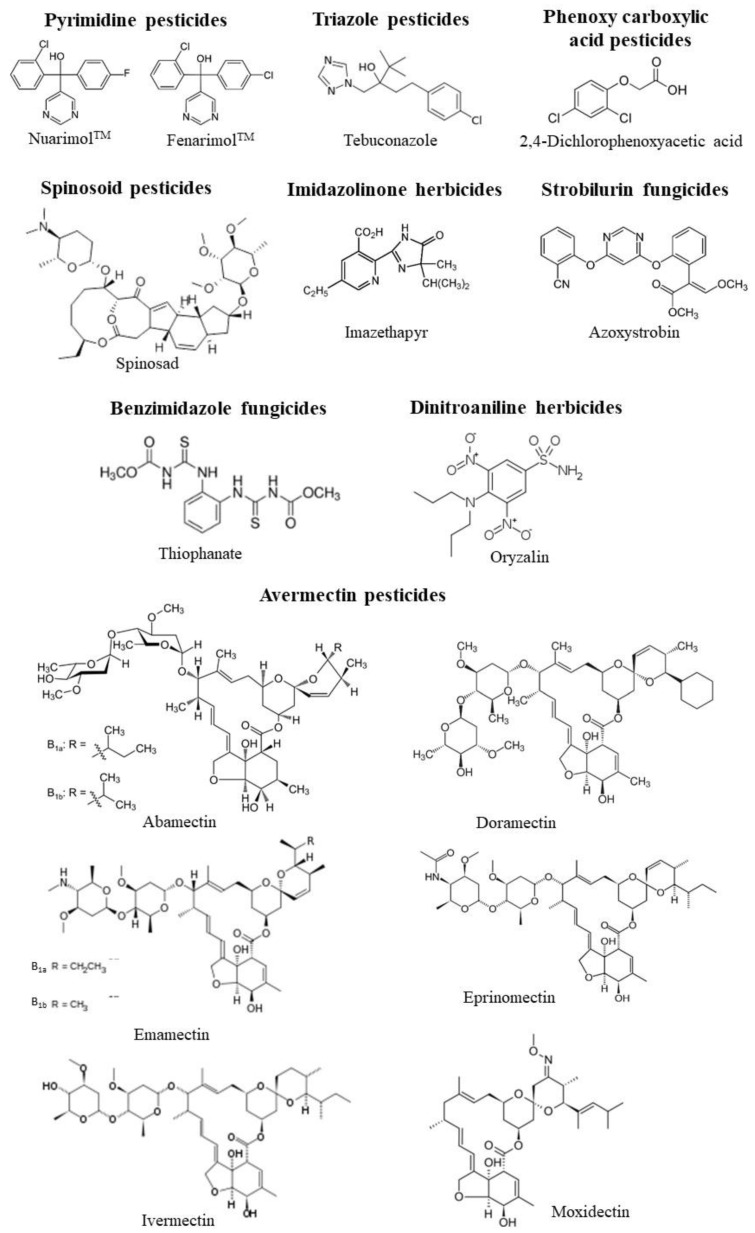
The chemical structure of the pesticides analyzed in Table 1 belonging to pyrimidines, triazole pesticides, phenoxycarboxylic acid pesticides, spinosoid pesticides, imidazoline herbicides, strobilurin fungicides, benzimidazole fungicides, dinitroaniline herbicides, avermectins.

The in vitro effects of different classes of pesticides on CA activity form different species are summarized in Table 1, which reports the IC_50_ values and the Ki values when present. The chemical structure of all the pesticides analyzed in Table 1 is shown in Figure 1 and Figure 2.

Organophosphates, which are esters of phosphoric acid and represent one of the most widely used classes of pesticides known for their neurotoxic effect through cholinesterase inhibition, showed a clear inhibitory potential on CA activity. Their IC_50_ values ranged from nanomolar to millimolar, showing high variability among specific pesticides of the same class, among species and tissues. For example, bovine CA erythrocytes showed comparable IC_50_ values for dichlorvos, methamidophos, methylparathion, included in the micromolar range, while the IC_50_ for chlorpyrifos was one order of magnitude higher, indicative of a lower sensitivity [24]. This result suggests the presence of variability among specific pesticides of the same organophosphate class on the CA inhibition. Moreover, the IC_50_ values measured for methylparathion, one of the most used organophosphate pesticide, on erythrocyte CA in different species showed different values ranging from 2.1 µM in bovine CA [24] to millimolar values in *Cyprinus carpio*, *Salmo gairdnerii*, *Barbus barbus*, *Diplodus vulgaris* [26], and even stimulation in *Capra hircus* [27]. This suggests species-specific variability in the sensitivity of CA activity to organophosphates. Moreover, the variability was also tissue-specific. In fact, in the same species, the same organophosphate pesticide can exert different inhibitory effects on CA from different tissues, as in the case of diazinon, whose IC_50_ value was 0.267 µM for the gill CA and 6.84 mM for the erythrocyte CA in the fish *Oncorynchus mykiss* [22]. 

Carbamates, which are structurally and mechanistically similar to organophosphate (OP) but are derived from a carbamic acid, showed a very high inhibitory potential against CA activity with IC_50_ values ranging from nanomolar to submillimolar. In the case of carbamate, it was also possible to observe variability among specific pesticides. For example, IC_50_ values for carbaryl and carbofran on *Apis mellifera* CA were comparable, being both in the nanomolar range, and the same values were also observed for the two thiocarbamates propineb and thiram on the muscle and gills CA of the fish *Trachurus trachurus* [34,35]. On the other hand, the IC_50_ value for propoxur was one order of magnitude higher [31]. Moreover, the dithiocarbamates maneb and propineb, tested on erythrocyte CA of the fish *Acipenser gueldenstaedti*, showed a higher inhibitory potential than carbaryl, as indicated by the lower IC_50_ value in the micromolar range [28]. As regards the comparison of IC_50_ values for the same carbamate pesticide on the same tissue in different species, the carbaryl IC_50_ values on *Bos taurus* and *Acipenser gueldenstaedti* erythrocyte CA were some order of magnitude different [28,32].

Also, pyrethroids, which constitute the majority of commercial household insecticides similar in the structure to the natural pyrethrins, proved to be potent CA inhibitors. Data available come from the three main pyrethroid used: deltamethrin, cypermethrin, and cyhalothrin [22,23,24,33,34,35]. Their IC_50_ values ranged from nanomolar to submillimolar values. In general, the three pesticides showed a similar behavior on CA from different tissues of three fish species analyzed such as *Oncorynchus mykiss*, *Cyprinus carpio*, and *Dicentrarchus labrax*, with great sensitivity in the liver, muscle, kidney, and brain CA, and lower sensitivity in the erythrocyte CA. The lower sensitivity of erythrocyte CA in fish towards pyrethroid pesticides was also confirmed in bovine erythrocyte CA [22,23,24,33]. Among pyrethroids, the highest sensitivity to inhibition was observed for muscle and gills CA of the fish *Trachurus thracurus* with a IC_50_ value in the nanomolar range [34,35]. 

Other classes of pesticides showed a significant inhibitory potential against CA, such as organophosphonates with glyphosate isopropylamine particularly effective on CA from sheep stomach [25], dinitrophenol pesticides, with dinocap particularly effective on the liver, brain, muscle, and kidney CA of the fish *Oncorhynchus mykiss* [33], triazine and triazole pesticides, highly effective on *Apis mellifera* CA [31] with IC_50_ values in the nanomolar range, imidazolinone herbicides, tested against human erythrocyte CA [30]. The tetrazine pesticide clofentezine was a potent inhibitor of *Trachurus trachurus* muscle and gill CA with nanomolar IC_50_ values [34,35]. Two classes of fungicides, strobilurin fungicides and benzimidazole fungicides, proved to be potent CA inhibitors with IC_50_ values in the nanomolar range in the muscle and gill CA of the fish *Trachurus trachrus* [34,35]. Avermectin pesticides and dinitroaniline herbicides were also able to strongly inhibit human CA [36,37]. Avermectins, generated as fermentation products by the soil actinomycete *Streptomyces avermitilis*, are macrocyclic lactonic compounds naturally occurring, with potent anthelmintic and insecticidal properties, widely utilized for the protection of animals and crops. Their known mechanism of action is based on the blocking on the transmission of electrical activity in invertebrate nerve and muscle cells, mostly by enhancing the effects of glutamate at the invertebrate-specific glutamate-gated chloride channel [38]. They were tested against CA II from bovine erythrocyte showing a high inhibitory potential with IC_50_ ranging from 14 to 21 nM [36] comparable to the acetazolamide IC_50_ value (IC_50_ = 24 nM), known specific CA inhibitor, determined on CA II from bovine erythrocytes. 

Moreover, dinitroaniline herbicides showed great inhibitory potential, with oryzalin particularly effective on the human CA I, II, and XIV isoforms [35].

### 2.2. Comparison among Species

Data reported in Table 1 allow also a comparison in the sensitivity to pesticides of CA from different species. 

In the case of low vertebrates, rainbow trout (*Oncorynchus mykiss*) was the most investigated species. It showed the highest sensitivity to a wide variety of pesticides compared to other fish species. Rainbow trout CA activity showed submicromolar IC_50_ values for carbamates, pyrethroids, dinitrophenol pesticides, and diazinon (organophosphate pesticide) in different tissues of the animal, such as gills, brain, liver, and muscle [22,33].

As regards mammals, bovine erythrocyte CA was one of the most investigated isoforms. Bovine CA was tested on the most utilized classes of pesticides, such as organophosphates, carbamates, pyrethroids, and avermectins. Among these classes, the lowest IC_50_ values were observed for avermectins (in the nanomolar range) followed by carbamates with an IC_50_ of 0.10 µM for carbaryl [32]. The sensitivity to organophosphate was sensibly lower, ranging from 2.150 µM for methylparathion to 84.12 µM for chlorpyrifos. The sensitivity to pyrethroids was in the micromolar range, with values ranging from 2.336 µM for cyhalothrin to 28.442 µM for cypermethrin.

In the case of humans, erythrocyte CAI and CAII showed IC_50_ in the micromolar range for several classes of pesticides such as organophosphonate, carbammate, pyrethroid, phenoxy carboxylic acid pesticides, and imidazolinone herbicides. Moreover, several human CA isoforms (CAI, II, IV, and XIV) showed a very high sensitivity (K_i_ values in the nanomolar range) towards oryzalin [37], a dinitroaniline herbicide widely employed for the control of annual grasses on a variety of food crops, and under considerations for the treatment of helminthiasis [39]. 

Among invertebrates, the only data available come from *Apis mellifera*, which showed very high sensitivity to several classes of pesticides, such as carbamate, triazine pesticides, and triazole pesticides showing submicromolar (10^−2^–10^−1^ micromolar) values, which reached the nanomolar range for carbaryl, carbofuran, and tebuconazole [31].

In order to explain the great variability observed in the in vitro sensitivity of CA from different species and different tissues to pesticides, it is possible to argue that structural differences in CA isoforms could produce different interactions between the protein and the specific pesticide molecule and in turn different inhibitory responses. 

### 2.3. Mechanisms of Action

**Figure 3:**
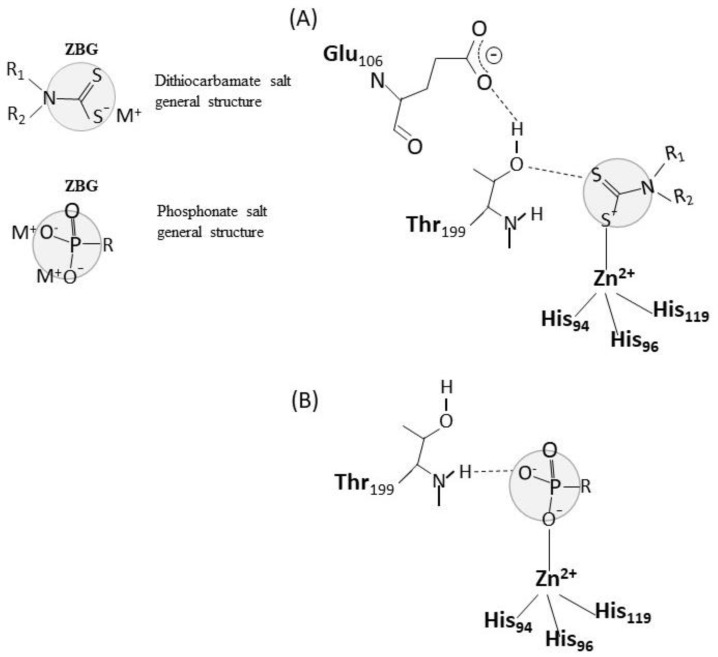
Dithiocarbamates and phosphonates as zinc binders of CA. The general structure of dithiocarbamates and phosphonates are reported with highlighted the zinc binding group (ZBG) of the molecules. The interaction of dithiocarbamates and phosphonates with the CA active site is shown in (**A**) and (**B**), respectively, according to [40,41,42].

In vitro exposure experiments provided information about the interaction of pesticides with the CA molecule, allowing to argue about possible toxic mechanisms of action, although to date, the mechanisms by which pesticides can inhibit CA have been poorly investigated. In general, the inhibitors of CA are distinguished in five different classes: (a) zinc binders, like the specific inhibitors sulphonamides, which coordinate to the zinc ion of the active site, with the metal in tetrahedral or trigonal bipyramidal geometries, (b) inhibitors that anchor to the zinc-coordinated water molecule/hydroxide ion, (c) inhibitors which occlude the entrance to the active site cavity, (d) compounds which bind out of the active site cavity, (e) compounds for which the inhibition mechanism is not known [6]. Dithiocarbamates and also phosphonate are known zinc binders [40,41,42], which bind as anions to the Zn^2+^ of the CA active site as demonstrated by crystallographic analysis. As shown in Figure 3, the zinc-binding groups of these molecules are coordinated to the Zn^2+^ metal ion, which in turn is bound to His94, His96, and His119, and makes hydrogen bonds with the residues Thr199–Glu106, which function as a gatekeeper and are conserved in all α-CAs. In the case of dithiocarbamates, a sulfur atom is involved in the coordination of the zinc binding group to the zinc ion, and a second sulfur atom is involved in an hydrogen bond with the OH of Thr 199 [41]. In the case of phosphonates, one of the oxygen atoms of the phosphonate moiety is coordinated to the zinc ion, while another oxygen atom is hydrogen-bonded to the backbone NH of Thr 199 [42] (Figure 1). In any case, the hydrogen bond with Thr199 stabilizes the adducts. Therefore, it is plausible that the thiocarbamate pesticides maneb and propineb or the organophosphonate pesticide glyphosate isopropylamine, which have been demonstrated to efficiently inhibit CA (see Table 1) exert their inhibitory activity as zinc binders, according to the general mechanism of their own chemical class. 

Moreover, similar behavior has also been argued for avermectins by Kose et al. [36], thank the presence of electronegative atoms in the structure of these compounds that could enable these molecules to bind the Zn^2+^ atom in the active site. This could explain the high inhibitory potential of avermectins, as expressed by their low IC_50_ values.

The dinitroaniline herbicide oryzalin has been demonstrated by fluorescence-based thermal shift assay and isothermal titration calorimetry to bind 12 human CA isoforms (I, II, IV, VA, VB, VI, VII, IX, XII, XIII, and XIV) with affinity in the same range as acetazolamide [37]. Besides, this compound proved to be a potent CA activity inhibitor with IC_50_ in the nanomolar range for human CA I, II, IV, and XIV. This compound contains a primary sulfonamide group, which can explain the high inhibitory potential of this molecule. Therefore, the mechanisms of action of oryzalin on human CAs can be referred to as the general mechanisms of action of sulfonamides as zinc binders.

To the best of our knowledge, no more information is available in the literature about the molecular mechanisms of action of other classes of pesticides on CA activity, and it is not possible to exclude that other types of inhibition can be involved for the other classes of pesticides considering the chemical diversity of the molecules involved. 

Moreover, it has to be mentioned that in one case [27], the case of methyl parathion on the sheep stomach CA activity in vitro, a stimulation was observed. In general, it is known that CA activators bind within the enzyme active cavity participating in the rate-determining step of the catalytic cycle, represented by the proton transfer between the active site and the environment [43]. Therefore, it is possible to hypothesize that this type of interaction can be involved in the in vitro stimulatory effect of methyl parathion on CA from the sheep stomach, and it is not possible to exclude that other not yet tested pesticides could act as CA activators on specific CA isoforms.

From all the in vitro studies to date available, it is possible to highlight the great number of in vitro toxicological evidence of the sensitivity of CA to pesticides. This evidence demonstrated the great potential of a number of pesticide classes to direct interact with CAs molecule and to inhibit the catalytic activity; at the same time it suggests the need to address new studies to clarify the mechanisms of interaction between pesticides and CA isoforms in humans and wildlife, and the molecular aspects underlying the variability observed.

## 3. In Vivo Sensitivity of CA Activity to Pesticides in Animals 

In vitro exposure experiments are useful for an early toxicological assessment and for establishing sensitivity classifications among different types of pesticides. Moreover, they are useful for predicting possible effects in vivo and in the field. However, in vitro exposure does not provide information about absorption, distribution, metabolism, and excretion of compounds. Therefore, in vivo studies were considered for a more realistic evaluation of the sensitivity of CA to pesticide exposure.

Table 2 summarizes the results of the in vivo CA sensitivity to pesticides from available studies. As it is possible to observe, all the in vivo experiments have been performed on fish, and in most cases, they confirm the inhibitory effects observed in vitro.

Deltamethrin, which is one of the most effective pesticides *in vitro* on fish, showed a time- and dose-dependent inhibition on rainbow trout (*Oncorynchus mykiss*) gills CA with the lowest concentration tested, 0.25 μg/L, statistically effective after 72 h, and the highest concentration tested (2.5 μg/L) statistically effective after 12 h [22]. These concentrations are included in the LC_50_ range of values reported in fish for deltamethrin [33]. Other authors [33] confirmed the dose- and time-dependent in vivo inhibition of deltamethrin on *Oncorynchus mykiss* CA also for other organs such as muscle, kidney, and liver, with the maximum effect observed in muscle and the lowest effect observed in the liver. 

Glyphosate was tested in vivo on zebrafish (*Danio rerio*) embryos, which are used as animal models of choice for vertebrate developmental studies [46]. The authors demonstrated a dose-dependent CA inhibition, with a significant effect at the lowest concentration tested of 1 mg/L. 

The herbicide atrazine exerted an in vivo inhibitory effect on gill CA of the neotropical freshwater fish *Prochilodus lineatus* showing a significant effect after 14 days of exposure at the higher concentration tested of 25 µg/L [47].

Among organophosphates, chlorpyrifos and parathion were in vivo tested. Chlorpyrifos was able to induce in vivo time- and dose-dependent inhibition on gill and liver CA in rainbow trout (*Oncorhynchus mykiss)* tested for 24–96 h at concentrations lower than the LC_50_ value of the species [45]. Parathion was tested on the euryhaline teleost *Oreochromis hornorum* at concentrations ranging from 8.3 µg/L, corresponding to 1/50 of the LC_50_ value, to 20.8 µg/L, corresponding to 1/20 of the LC_50_ value respectively [44]. The in vivo effect of the pesticide on gill and mesonephron CA was studied in parallel with changing salinity and a dose- and salinity-dependent multifaceted response was observed. In the gills, the pesticide exposure induced an increase in the CA activity at lower salinity, while at higher salinity a dose-dependent behavior was observed, with inhibition at lower concentrations and induction at higher concentrations. Also in mesonephron the response was dose- and salinity-dependent: the pesticide caused induction at 8.3 µg/L and inhibition at 10.4 µg/L at high salinity while the other concentration tested were ineffective, at lower salinity inhibition was observed at 10.4 µg/L and 13.9 µg/L, while the other concentrations tested were ineffective [44]. These results suggest that the in vivo effect of methyl parathion on *Oreochromis hornorum* gills and mesonephron CA can be made more complex by exposure to other stressful conditions and that involvement of the pesticide in the mechanisms underlying the expression of the enzyme cannot be excluded. 

Although in vivo studies are far less numerous than the in vitro studies and more research is required in this field, they highlight that CA is an in vivo target of the effect of several classes of pesticides allowing to more realistically predict possible effect in the field.

## 4. Perspectives for Pesticide Biomarker Development

The great number of in vitro studies on the sensitivity of CAs to pesticides in a variety of species and tissues provide information on the remarkable capability of numerous pesticide molecules to interact with CA and to dose-dependently inhibit the catalytic activity. On the other hand, in vivo studies, despite their few numbers compared to in vitro studies, describe the capacity of some pesticides to reach in vivo effective concentrations for CA inhibition in several body tissues and allow to know negative health effects related to CA activity depression. 

Although further studies are needed to more deeply clarify several aspects related to pesticide inhibition of CA activity in different species, such as binding affinity, mechanisms of inhibition, in vivo effects on CA activity and expression, the relationship between CA pesticide alteration and health effects, the data available opens new perspectives for the development of CA-based pesticide biomarkers suitable for application in several fields from environmental to human biomonitoring.

### 4.1. Potential Biomarker of Effect 

It is known that CA plays a key role in a number of physiological processes in animals. CA facilitates the transport and subsequent excretion of CO_2_ through the respiratory surfaces, being involved in any step of the overall process, including the site of CO_2_ production at the peripheral tissue level, the circulating red blood cells and the respiratory surface [48]. In the acid-base-regulation, CA regulates the production of bicarbonate, which represents the universal physiological buffer, and, at the same time, CA produces or sequesters protons [49]. CA determines the HCO_3_^-^ local concentration during fluid and HCO_3_^-^ secretion, influencing the activity of all transporters involved in this process fundamental for the regulation of systemic and cellular pH, cell volume, and solubilization of macromolecules [50]. CA activity is also required for a number of metabolic processes such as gluconeogenesis, urea biosynthesis, and lipogenesis [51], and some CA isoforms have been related to some signal transduction pathways [52]. Human CAIII and CAVII are included in the antioxidant defense system of the body [5,53]. CA in the digestive gland of mussels, widely utilized as sentinel organisms in environmental biomonitoring, is functionally related to lysosomal activation following pollution exposure [54,55].

Considering the key physiological roles played by CA in animals, it is reasonable to hypothesize that any alteration of CA activity by pesticide exposure could represent a threat to the health status of the organism. Although very little information is available to date on the direct relationships between pesticide exposure, CA inhibition and health status impairment, some experimental evidence highlights the alteration of some physiological functions caused by CA inhibition induced by pesticide exposure. For example, Paulino et al. [47] demonstrated plasma osmolality and Na^+^ and Cl^−^ concentration changes associated with CA inhibition induced by atrazine exposure in the neotropical fish, *Prochilodus lineatus*. Sulukan et al. [46] found CA inhibition in zebrafish embryos following glyphosate exposure associated with an increased ROS production at the level of the gills as a consequence of the decreased CO_2_ extraction and, in turn, respiratory acidosis. Although further research is required in this field, the experimental evidence suggests the possible relevance of CA alterations as pesticide biomarkers of effect. There is still a lot of work to be done, but the in vitro and in vivo results to date available could pave the way for future studies focusing on the most sensitive bioindicator species and on the most effective CA inhibitor pesticides. 

Moreover, in humans it is known that CAIII and CA VII are related to the protection of the cell from oxidative stress damaging effects, thus participating in the intracellular antioxidant defense system [5]. They can operate as scavengers of reactive species through reactive sulfhydryl groups present in their high number of cysteine residues [53]. The involvement of these proteins in the response to oxidative stress is also supported by the observation that they undergo glutathionylation, the reversible binding of glutathione to thiolate anions of cysteine residues [5]. These two enzymes are both highly expressed in tissues characterized by high oxygen consumption rates, such as skeletal muscle, liver, and brain [5,56], and their presence has been demonstrated also in other tissues, including erythrocytes for CAIII in humans [57,58]. It is known that CAIII glutathiolation is increased under acute oxidative stress [59,60] as an early response to oxidative insult and essential component of cellular antioxidant defense. A number of pesticides are known to induce oxidative stress by overproduction of reactive species and alterations of antioxidant defenses in wildlife [45,61,62] and humans [63,64,65]. Therefore, these considerations open the perspective for possible development of CA III glutathionilation in the blood as an effect biomarker of the oxidative stress induced by the exposure to pesticides, suitable for application in occupational and environmental medicine.

### 4.2. Potential Biomarker of Exposure

Protein adducts have been recently affirmed as a useful marker of biologically effective dose, which faithfully reflects external chemical exposure [66]. In human biomonitoring of pesticide exposure, the most known protein adducts to date are represented by organophosphate-adducted serine esterases [67], as an alternative approach to the standard method in use for organophosphate exposure biomonitoring represented by cholinesterase inhibition assessment [15]. In the last years, there is increasing interest in the analysis of protein adducts as biomarkers of pesticide exposure since adducts can have longer half-lives than parent compounds or metabolites in body fluids, are an expression of the interaction that the active pesticide can establish with target proteins, and their measure is highly sensitive. 

The data on in vitro inhibition of CA activity by several pesticides in both humans and wildlife provide knowledge on the high potential of interaction of CA with numerous pesticide molecules and in turn on the high potential of this enzyme for adduct formation with pesticide molecules. This opens new perspectives for the study of CA pesticides adducts as exposure biomarker to pesticides for application in human and environmental biomonitoring. 

## 5. Conclusions

In conclusion, the knowledge available to date on the sensitivity of CA to pesticides in humans and wildlife opens new perspectives for the promising development of novel sensitive CA based pesticide biomarkers. The analysis of the available data has revealed key areas in which further research is needed in this field, but at the same time has highlighted the ability of a number of pesticide molecules to directly interact with the enzyme in humans and wildlife and to inhibit CA activity with possible alterations of key physiological functions. This offers a wide range of perspectives for the development of novel sensitive biomarkers (either exposure or effect biomarkers) suitable to be applied in several areas of interest from human biomonitoring in occupational and environmental medicine to environmental monitoring on nontarget species.

## Figures and Tables

**Table 1:** In vitro effect of several classes of pesticides on CA activity from several species.

Pesticides	IC_50_ (µM)	Ki (µM)	Inhibition Type	Species	Tissue	Ref.
***Organophosphate***						
Diazinon	0.267	n.d.	n.d.	*Oncorynchus mykiss*	gills	[22]
	6840	n.d.	n.d.	*Oncorynchus mykiss*	erythroytes	[23]
	3920	n.d.	n.d.	*Cyprinus carpio*	erytrocytes	[23]
Dichlorvos	23.19	14.17	n.d.	*Dicentrarchus labrax*	liver	[24]
	10.178	5.872	n.d.	*Bos taurus*	erytrocytes	[24]
	2.690	3.700 ± 1.670	non competitive	*Ovis aries*	stomach	[25]
Methamidophos	38.73	26.16	n.d.	*Dicentrarchus labrax*	liver	[24]
	2.129	1.636	n.d.	*Bos taurus*	erytrocytes	[24]
Chlorpyrifos	2.640	2.175	n.d.	*Dicentrarchus labrax*	liver	[24]
	84.12	53.28	n.d.	*Bos taurus*	erytrocytes	[24]
Methyl parathion	0.850	0.376	n.d.	*Dicentrarchus labrax*	liver	[24]
	2.150	1.174	n.d.	*Bos taurus*	erytrocytes	[24]
	620.0	n.d.	n.d.	*Scorpaena porcus*	erytrocyte	[26]
	2900	n.d.	n.d.	*Diplodus vulgaris*	erytrocyte	[26]
	1770	n.d.	n.d.	*Salmo gairdnerii*	erytrocyte	[26]
	2450	n.d.	n.d.	*Barbus barbus*	erytrocyte	[26]
	3190	n.d.	n.d.	*Diplodus vulgaris*	erytrocyte	[26]
	stimul.	n.d.	n.d.	*Capra hircus*	erytrocytes	[27]
Parathion	102	n.d.	n.d.	*A. gueldenstaedti*	erythrocytes	[28]
Dimetoate	520	n.d.	n.d.	*Barbus grypus*	gills	[29]
***Organophosphonate***						
Glyphosate isopropylamine	0.150	0.319 ± 0.067	non competitive	*Ovis aries*	stomach	[25]
	15.2	n.d.	n.d.	*Homo sapiens* (CA I)	erytrocyte	[30]
	62.8	n.d.	n.d.	*Homo sapiens* (CA II)	erytrocyte	[30]
***Carbamate pesticides***						
Propoxur	0.420	n.d.		*Oncorynchus mykiss*	gills	[22]
	0.032	n.d.	n.d.	*Apis mellifera*	whole animal	[31]
Carbaryl	0.003	n.d.	n.d.	*Apis mellifera*	whole animal	[31]
	0.100	n.d.	n.d.	*Bos taurus*	erytrocytes	[32]
	398	n.d.	n.d.	*A. gueldenstaedti*	erythrocytes	[28]
Carbofuran	0.009	n.d.	n.d.	*Apis mellifera*	whole animal	[31]
Mancozeb	0.368	n.d.	n.d.	*Oncorhynchus mykiss*	muscle	[33]
	0.505	n.d.	n.d.	*Oncorhynchus mykiss*	brain	[33]
	0.151	n.d.	n.d.	*Oncorhynchus mykiss*	liver	[33]
	0.306	n.d.	n.d.	*Oncorhynchus mykiss*	kidney	[33]
Propamoarb	90.4	n.d.	n.d.	*Homo sapiens* (CA I)	erytrocyte	[30]
	62.0	n.d.	n.d.	*Homo sapiens* (CA II)	erytrocyte	[30]
Maneb (dithiocarbam.)	18.0	n.d.	n.d.	*A. gueldenstaedti*	erythrocytes	[28]
Propineb (dithiocarbam.)	16.0	n.d.	n.d.	*A. gueldenstaedti*	erythrocytes	[28]
	0.0094	0.0098 ± 0.0048	uncompetitive	*Trachurus trachrus*	muscle	[34]
	0.0084	0.0111 ± 0.0050	uncompetitive	*Trachurus trachrus*	gills	[35]
Thiram (dithiocarbam.)	0.0058	0.0057 ± 0.0023	uncompetitive	*Trachurus trachrus*	muscle	[34]
	0.0032	0.0043 ± 0.0020	uncompetitive	*Trachurus trachrus*	gills	[35]
**Pyrethroid pesticides**						
Deltamethrin	0.137	n.d.	n.d.	*Oncorynchus mykiss*	gills	[22]
	0.097	n.d.	n.d.	*Oncorynchus mykiss*	liver	[33]
	0.237	n.d.	n.d.	*Oncorynchus mykiss*	muscle	[33]
	0.161	n.d.	n.d.	*Oncorynchus mykiss*	kidney	[33]
	0.160	n.d.	n.d.	*Oncorynchus mykiss*	brain	[33]
	14.8	n.d.	n.d.	*Oncorynchus mykiss*	erytrocytes	[23]
	470	n.d.	n.d.	*Cyprinus carpio*	erytrocytes	[23]
	0.0085	0.0076 ± 0.0011	uncompetitive	*Trachurus trachrus*	muscle	[34]
	0.012	0.0011 ± 0.0031	uncompetitive	*Trachurus trachrus*	gills	[35]
Cypermethrin	0.460	n.d.	n.d.	*Oncorynchus mykiss*	gills	[22]
	1.248	0.832	n.d.	*Dicentrarchus labrax*	liver	[24]
	28.440	16.17	n.d.	*Bos taurus*	erythrocytes	[24]
	0.256	n.d.	n.d.	*Oncorynchus mykiss*	liver	[33]
	0.700	n.d.	n.d.	*Oncorynchus mykiss*	muscle	[33]
	0.220	n.d.	n.d.	*Oncorynchus mykiss*	kidney	[33]
	0.491	n.d.	n.d.	*Oncorynchus mykiss*	brain	[33]
Cyhalothrin	1.895	1.074	n.d.	*Dicentrarchus labrax*	liver	[24]
	2.336	1.863	n.d.	*Bos taurus*	erythrocytes	[24]
	605	n.d.	n.d.	*Oncorynchus mykiss*	erythrocytes	[23]
	686	n.d.	n.d.	*Cyprinus carpio*	erythrocytes	[23]
***Dinitrophenol pesticides***						
Dinocap	0.102	n.d.	n.d.	*Oncorhynchus mykiss*	liver	[33]
	0.263	n.d.	n.d.	*Oncorhynchus mykiss*	muscle	[33]
	0.199	n.d.	n.d.	*Oncorhynchus mykiss*	kidney	[33]
	0.190	n.d.	n.d.	*Oncorhynchus mykiss*	brain	[33]
2,4-Dichlorophenol	240	n.d.	n.d.	*A. gueldenstaedti*	erythrocytes	[28]
***Triazine and tetrazine pesticides***						
Simazine	0.0273	n.d.	n.d.	*Apis mellifera*	whole animal	[31]
Atrazine	0.0165	n.d.	n.d.	*Apis mellifera*	whole animal	[31]
Clofentezine	0.0038	0.0023 ± 0.0002	competitive	*Trachurus trachrus*	muscle	[34]
	0.0035	0.0053 ± 0.0022 *	competitive	*Trachurus trachrus*	gills	[35]
***Pyrimidine pesticides***						
Nuarimol^TM^	352	n.d.	n.d.	*Capra hircus*	erytrocyte	[27]
	380	n.d.	n.d.	*Cyprinus carpio*	erytrocyte	[26]
	200	n.d.	n.d.	*Scorpaena porcus*	erytrocyte	[26]
	280	n.d.	n.d.	*Barbus barbus*	erytrocyte	[26]
	230	n.d.	n.d.	*Salmo gairdnerii*	erytrocyte	[26]
	380	n.d.	n.d.	*Diplodus vulgaris*	erytrocyte	[26]
Fenarimol^TM^	924	n.d.		*Capra hircus*	erytrocyte	[27]
	550	n.d.	n.d.	*Cyprinus carpio*	erytrocyte	[26]
	180	n.d.	n.d.	*Scorpaena porcus*	erytrocyte	[26]
	590	n.d.	n.d.	*Barbus barbus*	erytrocyte	[26]
	510	n.d.	n.d.	*Salmo gairdnerii*	erytrocyte	[26]
	370	n.d.	n.d.	*Diplodus vulgaris*	erytrocyte	[26]
***Triazole pesticides***						
Tebuconazole	0.003	n.d.		*Apis mellifera*	wholeanimal	[31]
***Phenoxy carboxylic acid pesticides***						
2,4-D	2040	n.d.	n.d.	*Capra hircus*	erytrocyte	[27]
	61.7	n.d.	n.d.	*Homo sapiens* (CA I)	erytrocyte	[30]
	66.0	n.d.	n.d.	*Homo sapiens* (CA II)	erytrocyte	[30]
	650	n.d.	n.d.	*Scorpaena porcus*	erytrocyte	[26]
	2720	n.d.	n.d.	*Cyprinus carpio*	erytrocyte	[26]
	1730	n.d.	n.d.	*Barbus barbus*	erytrocyte	[26]
	1260	n.d.	n.d.	*Salmo gairdnerii*	erytrocyte	[26]
	2670	n.d.	n.d.	*Diplodus vulgaris*	erytrocyte	[26]
***Spinosoid pesticides***						
Spinosad	410	n.d.	n.d.	*Barbus grypus*	gills	[29]
***Imidazolinone herbicides***						
Imazethapyr	93.0	n.d.	n.d.	*Homo sapiens* (CA I)	erytrocyte	[30]
	46.3	n.d.	n.d.	*Homo sapiens* (CA II)	erytrocyte	[30]
***Strobilurin fungicides***						
Azoxystrobin	0.0301	0.0307 ± 0.0100 *	competitive	*Trachurus trachrus*	muscle	[34]
	0.0309	0.0139 ± 0.0032	competitive	*Trachurus trachrus*	gills	[35]
***Benzimidazole fungicides***						
Thiophanate	0.0705	0.0898 ± 0.0322	uncompetitive	*Trachurus trachrus*	muscle	[34]
	0.0367	0.0484 ± 0.0140	uncompetitive	*Trachurus trachrus*	gills	[35]
***Avermectin pesticides***						
Abamectin	0.0144	0.0097 ± 0.0019 **	n.d.	*Bos taurus*	erytrocytes	[36]
Doramectin	0.0146	0.0174 ± 0.0048 **	n.d.	*Bos taurus*	erytrocytes	[36]
Emamectin	0.0187	0.0020 ± 0.0095 **	n.d.	*Bos taurus*	erytrocytes	[36]
Eprinomectin	0.0146	0.0134 ± 0.0025 **	n.d.	*Bos taurus*	erytrocytes	[36]
Ivermectin	0.0145	0.0164 ± 0.0053 **	n.d.	*Bos taurus*	erytrocytes	[36]
Moxidectin	0.0208	0.0177 ± 0.0037 **	n.d.	*Bos taurus*	erytrocytes	[36]
***Dinitroaniline herbicides***						
Oryzalin	n.d.	0.029	n.d.	*Homo sapiens* (CA I)	reconbinant	[37]
Oryzalin	n.d.	0.008	n.d.	*Homo sapiens* (CA II)	reconbinant	[37]
Oryzalin	n.d.	0.195	n.d.	*Homo sapiens* (CAIV)	reconbinant	[37]
Oryzalin	n.d.	0.002	n.d.	*Homo sapiens* (CA XIV)	reconbinant	[37]

* The values may be incorrect because the relationship between K_i_ and IC_50_ does not obey the Cheng–Prusoff equation; ** The K_i_ values were obtained by an esterase assay with 4-nitrophenylacetate as substrate, while the corresponding IC_50_ values were obtained by the CO_2_ hydration reaction; 2,4-d (an abbreviation of 2,4-Dichlorophenoxyacetic acid).

**Table 2:** In vivo effect of pesticides on CA activity from several species.

Pesticides	Species	Tissue	Concentration Tested	Duration of Exposure	Effect Observed on CA	Ref
**Organophosphate** **pesticides**						
Parathion	*Oreochromis hornorum*	Gills, mesonephron	8.3, 10.4, 13.9, 20.8 µg/L	72 h	Dose- and salinity-dependent induction/inhibition	[44]
Chlorpyrifos	*Oncorhynchus mykiss*	Gills, liver	2.25, 4.5, 6.75 μg/L	24, 48, 72, 96 h	Dose- and time-dependent inhibition	[45]
**Organophosphonate** **pesticides**						
Glyphosate	*Daino rerio*	Whole embrios	1, 5, 10 and 100 mg/L	96h	Dose- and time-dependent inhibition	[46]
**Pyrethroid** **pesticides**						
Deltamethrin	*Onchorynchus mykiss*	Gills	0.25 μg/L, 1 μg/L, and 2.5 μg/L	6, 12, 24, 48,72 h	Dose- and time-dependent inhibition	[22]
	*Onchorynchus mykiss*	Muscle, liver, kidney	0.25 μg/L, 1 μg/L, and 1 μg/L	6, 12, 24, 48 h	Dose- and time-dependent inhibition	[33]
**Triazine** **pesticides**						
Atrazine	*Prochilodus* *lineatus*	Gills	2, 10, 25 μg/l	48 h and 14 days	Inhibition after 14 days	[47]
